# Prognostic significance of IMMT expression in surgically‐resected lung adenocarcinoma

**DOI:** 10.1111/1759-7714.13200

**Published:** 2019-10-03

**Authors:** Yasuhiro Hiyoshi, Yuichi Sato, Masaaki Ichinoe, Ryo Nagashio, Daisuke Hagiuda, Makoto Kobayashi, Seiichiro Kusuhara, Satoshi Igawa, Kazu Shiomi, Naoki Goshima, Yoshiki Murakumo, Makoto Saegusa, Yukitoshi Satoh, Noriyuki Masuda, Katsuhiko Naoki

**Affiliations:** ^1^ Department of Respiratory Medicine, School of Medicine Kitasato University Sagamihara Japan; ^2^ Applied Tumor Pathology, Graduate School of Medical Sciences Kitasato University Sagamihara Japan; ^3^ Department of Pathology, School of Medicine Kitasato University Sagamihara Japan; ^4^ Department of Thoracic and Cardiovascular Surgery, School of Medicine Kitasato University Sagamihara Japan; ^5^ National Institute of Advanced Industrial Science and Technology Tokyo Bay Area Center Koto‐ku Japan

**Keywords:** IMMT, lung adenocarcinoma, immunohistochemistry, prognostic marker, TCGA database

## Abstract

**Background:**

Mitochondrial dysfunction contributes to many types of human disorders and cancer progression. Inner membrane mitochondrial protein (IMMT) plays an important role in the maintenance of mitochondrial structure and function. The aims of this study were to examine IMMT expression in lung adenocarcinoma and evaluate its correlation with clinicopathological parameters and patient prognosis.

**Methods:**

IMMT expression was immunohistochemically studied in 176 consecutive lung adenocarcinoma resection tissues, and its correlations with clinicopathological parameters were evaluated. Kaplan‐Meier survival analysis and Cox‐proportional hazards models were used to estimate the effect of IMMT expression on survival.

**Results:**

High‐IMMT expression was detected in 84 of 176 (47.7%) lung adenocarcinomas. Levels were significantly correlated with advanced disease stage (stage II and III; *P* = 0.024), larger tumor size (>3 cm; *P* = 0.002), intratumoral vascular invasion (*P* < 0.001), and poorer adenocarcinoma patient prognosis (*P* = 0.002). Based on 176 patients with adenocarcinoma, multivariate analysis revealed that IMMT expression was an independent predictor of poorer survival (HR, 1.99; 95% confidence interval [CI], 1.06–3.74; *P* = 0.031). Further, treating A549 cells derived from lung adenocarcinoma, with IMMT siRNA resulted in significantly decreased proliferation.

**Conclusion:**

Here, we first demonstrated that high‐IMMT expression is related to some clinicopathological parameters, and that its expression is an independent prognostic predictor of poorer survival in patients with lung adenocarcinoma; further studies are required to clarify the biological function of IMMT in lung adenocarcinoma. However, results suggest that this protein could be a novel prognostic indicator and therapeutic target.

## Key points

### Significant findings of the study

High IMMT expression is associated with poorer prognosis of patients with lung adenocarcinoma. Based on the TCGA database, high *IMMT* mRNA expression is also associated with poorer prognosis of patients with lung adenocarcinoma.

### What this study adds

Following IMMT‐knockdown with siRNA in A549 lung adenocarcinoma cells, we confirmed the role of the *IMMT* gene in tumor cell proliferation.

IMMT‐knockdown A549 lung adenocarcinoma cells also exhibited decreased proliferation.

## Introduction

Primary lung cancer is the leading cause of cancer‐related mortality worldwide. Despite advances in surgical techniques and traditional chemoradiotherapeutic modalities, the overall five‐year survival rate for lung cancer patients has only slightly improved over the last few decades, with the current five‐year survival being approximately 15%.^1^ Furthermore, non‐small cell lung cancer (NSCLC) accounts for approximately 80% of lung cancers, of which approximately 50% are adenocarcinomas. Thus, a further understanding of the tumorigenesis and biology of lung cancer might be useful for the development of novel prognostic markers or therapeutic targets in lung adenocarcinoma. We previously generated monoclonal antibodies to tumor‐associated proteins using lung cancer cells or tissues, termed the random immunization method.^2^, ^3^, ^4^ The present study describes one antibody, designated KU‐Lu‐10, which recognizes the inner membrane mitochondrial protein (IMMT) by immunoprecipitation and mass spectrometry (Supplementary Data S1 and Fig S1).

Mitochondria play an essential role in several cellular functions including growth, division, apoptosis, and energy metabolism. Therefore, mitochondrial dysfunction contributes to many types of human disorders and cancer progression.^5^ IMMT has been reported to be a mitochondrial protein that affects morphological structure and has a presumptive impact on mitochondrial function.^6^ Although little is known about the function of IMMT, alterations to this marker have been reported to be associated with different diseases including Down's syndrome,^7^ diabetic cardiomyopathy,^8^ and Parkinson's disease.^9^ However, the role of IMMT remains mostly unknown in cancers including NSCLC. To our knowledge, no report has been published concerning the relationship between IMMT expression and clinicopathological features and patient prognosis based on a large number of cancer cases including lung adenocarcinoma. Therefore, the aims of this study were to immunohistochemically examine IMMT expression in surgically‐resected lung adenocarcinoma and analyze its correlation with clinicopathological parameters and patient prognosis.

## Methods

### Patients and tissue specimens

A total of 176 consecutive adenocarcinoma patients who underwent complete resection from January 2002 to December 2005 at Kitasato University Hospital were included in this retrospective cohort study. Patients receiving preoperative chemotherapy and/or radiotherapy were excluded. Ten percent formalin‐fixed and paraffin‐embedded tissues were processed into 3 μm thick sections and stained with hematoxylin and eosin. The histological diagnosis was based on the criteria of the 2015 World Health Organization Classification of Lung and Pleural Tumors.^10^ Each patient was reassessed according to the seventh edition of the TNM classification.^11^ The clinical and pathologic parameters were retrospectively reviewed including age at surgical resection, sex, smoking habits, histological type, tumor differentiation, pathological TNM (p‐TNM) and stage, nodal status, intratumoral vascular invasion, intratumoral lymphatic invasion, pleural invasion, administration of adjuvant chemotherapy, viability status, and survival time after surgery. The viability status was determined based on whether or not adenocarcinoma‐related death occurred, and the survival time was defined as the duration from the date of surgery to the date of death, or the end of follow‐up. Cases in which death occurred due to other causes or those lost to follow‐up were treated as censored cases. The study was approved by the Ethics Committee of the Kitasato University School of Medicine (B16‐103) and followed the Declaration of Helsinki protocol. All patients were approached based on approved ethical guidelines, agreed to participate in this study, and could refuse entry and discontinue participation at any time. All participants provided written consent.

### Lung adenocarcinoma cell lines

A549 and LC‐2/ad cell lines derived from lung adenocarcinomas were purchased from the American Type Culture Collection (Rockville, MD, USA) and the RIKEN BioResource Center (Ibaraki, Japan), respectively. The cell lines were cultured in RPMI‐1640 medium (FUJIFILM Wako Pure Chemical, Osaka, Japan) supplemented with 10% fetal bovine serum (MP Biomedicals, Inc., Santa Ana, CA, USA), 100 U/mL of penicillin, and 100 μg/mL streptomycin (Thermo Fisher Scientific, Waltham, MA, USA) at 37°C in 5% CO_2_ and 95% humidified air. Subconfluent cells were harvested and washed three times with phosphate‐buffered saline without divalent ions (PBS‐) and stored at −80°C.

### Immunohistochemical staining using IMMT antibody

After deparaffinization in xylene and rehydration in a descending ethanol series and tap water, the sections were treated with 3% hydrogen peroxide for 10 minutes. Antigen‐retrieval was then performed by autoclaving samples for 10 minutes in 0.01 M citrate buffer (pH 6.0) with 0.1% tween 20. After blocking with 2% normal swine serum/tris‐buffered saline (0.01 M Tris‐HCl, pH 7.5, 150 mM NaCl) for 10 minutes, the sections were reacted with nondiluted hybridoma supernatant containing the anti‐IMMT antibody for two hours at 37°C. After rinsing in Tris‐buffered saline three times for five minutes each, samples were reacted with ChemMate ENVISION reagent (Dako, Glostrup, Denmark) for 30 minutes at room temperature (RT). They were subsequently visualized with Stable DAB solution (Invitrogen, Carlsbad, CA, USA) and counterstained with Mayer's hematoxylin. Negative controls were incubated in supernatant from Sp2/0‐Ag14 myeloma cells (RIKEN BRC Cell Bank, Ibaraki, Japan) instead of with the anti‐IMMT antibody.

### Evaluation of immunohistochemical staining

For IMMT, cytoplasmic staining in tumor cells was considered positive. The evaluation of the cell staining reactions was performed based on the immunoreactive score (IRS) as follows: IRS = SI (staining intensity) × PP (percentage of positive tumor cells). SI was defined as follows: 0, negative; one, weak; two, moderate; three, strong. PP was defined as follows: 0, negative; one, 1–10% positive tumor cells; two, 11%–50% positive tumor cells; three, 51%–80% tumor cells; four, >80% positive tumor cells. An IRS value ≥4 was considered as high‐IMMT expression, whereas an IRS value <4 was considered low‐IMMT expression. Two investigators (H.Y. and S.Y.) separately evaluated all specimens in a blinded manner. Discordant cases were reviewed and discussed until a consensus was obtained for each specimen.

### Survival analysis based on the TCGA database

*IMMT* mRNA expression data (RNA Seq V2 PSEM) from lung adenocarcinoma (*N* = 517) was downloaded from cBioPortal (http://www.cboportal.org/). We stratified *IMMT* mRNA expression into two groups based on the lower quartile. The cumulative survival of patients was estimated using the Kaplan‐Meier method, and significant differences in survival between the two groups with different *IMMT* mRNA expression was tested using the log‐rank test.

### Western blotting

Proteins were extracted from lung adenocarcinoma cell lines with lysis buffer (250 mM Tris‐HCl pH 6.8, 2% SDS, 10% glycerol, 1% β‐mercaptoethanol, 2 mM phenylmethylsulphonyl fluoride). Five micrograms of each protein sample were applied to a 10% polyacrylamide gel for SDS polyacrylamide gel electrophoresis. Separated proteins on polyacrylamide gels were transferred to 0.45 μm polyvinylidene difluoride membranes (Merck‐Millipore, Darmstadt, Germany) overnight with a constant voltage of 10 V with transfer buffer (100 mM Tris (hydroxymethyl) aminomethane, 200 mM glycine). After blocking with 0.5% casein for one hour, membranes were incubated with nondiluted hybridoma supernatant containing the anti‐IMMT antibody for two hours at RT and HRP‐conjugated anti‐mouse IgG antibody (Dako, Glostrup, Denmark) diluted 1000‐fold for 45 minutes at RT. After each incubation step, the membranes were washed three times with Tris (hydroxymethyl) aminomethane‐buffered saline (TBS) containing 0.1% Tween 20 (TBS‐T) for 5 minutes each. Immunoreactive bands on the membranes were developed with Immobilon Western Chemiluminescent HRP Substrate (Merck‐Millipore) and captured with the Cool Saver System (ATTO, Tokyo, Japan).

### Transfection of IMMT siRNA and proliferation, migration, and invasion assays

For siRNA transfection, four sequences of *IMMT* siRNA (Qiagen, Venlo, Netherlands) and negative control siRNA (Bioneer, Daejeon, Korea) were used. A total of 1 × 10^4^ A549 cells were cultured in a 24 well plate (Sumitomo Bakelite Co., Ltd., Tokyo, Japan). After 24 hours, cells were transfected with Lipofectamine RNAiMAX transfection reagent (Thermo Fisher Scientific). A final concentration of 50 nM, 125 nM, and 250 nM of each siRNA for the *IMMT* gene and negative control were used. For cell proliferation assays, 5 × 10^3^ cells were analyzed 24 to 72 hours after transfection. Viable cells were estimated with the Cell Titer 96 Aqueous One Solution Cell Proliferation Assay (Promega Corp., Madison, WI, USA) using a 96 well plate, following the manufacturer's instructions. For migration and invasion assays, 48 hours after transfection, cells were resuspended at 1 × 10^4^ cells per 0.3 mL in serum‐free RPMI‐1640 medium and added to the upper chambers of 24 well Transwell inserts (Corning, Corning, NY, USA). Inserts were coated with 50 μL of Matrigel (Corning) dissolved in serum‐free RPMI medium for the invasion assay or left uncoated for the migration assay, as previously described.^12^ Cells in three random high‐power fields were counted in triplicate. A Student's *t*‐test was used for statistical analysis.

## Statistical analysis

Continuous variables are presented as the median (range), whereas numerical variables are given as N (%). The relationships between IMMT expression and clinicopathological parameters were assessed based on the Pearson's χ^2^ test or Fisher's exact test, as appropriate. The cumulative survival of patients was estimated using the Kaplan‐Meier method, and the significance of survival differences between high‐ and low‐IMMT expression groups was tested by performing the log‐rank test. The five‐year cumulative survival probability was estimated using the life table method with the interval length set at one month. Multivariate analysis was performed by employing the Cox‐proportional hazards regression model to examine the interaction between IMMT expression and other clinicopathological variables and estimate the independent prognostic effect of IMMT on survival by adjusting for confounding factors. The conventional *P*‐values of 0.05 or less was used to determine the level of significance. All reported *P*‐values were two‐sided. Analyses were performed using SPSS version 23.0 software (SPSS; Chicago, IL, USA).

## Results

### Clinicopathological characteristics of patients

The clinicopathological characteristics of the patients are summarized in Table 1. A total of 94 men and 82 women were included, with ages ranging from 37 to 82 years (median, 69.5 years); of these individuals, 89 (50.6%) were smokers. The 176 cases consisted of 58 lepidic (33.0%), 23 acinar (13.1%), 64 papillary (36.4%), 11 micropapillary (6.2%), 13 solid (7.4%), and seven invasive mucinous (4.0%) adenocarcinomas. The overall follow‐up duration ranged from four to 129 months (median, 104 months). A total of 105 patients were alive at the end of the follow‐up, 51 patients had died of lung cancer, eight died from other causes, and 12 were lost to follow‐up. None of the eight patients had surgery‐related deaths. Of the 12 lost to follow‐up, all were lost due to discontinuing hospital attendance and could not be contacted. The follow‐up durations of the 12 patients lost to follow‐up ranged from 22 to 87 months (median, 66 months).

**Table 1:** Characteristics of patients with lung adenocarcinoma

Characteristics	Patients (*N* = 176)
Age	
Median age (range)	69.5 (37–82)
<65 years	94 (53.4)
≥65 years	82 (46.6)
Sex	
Male	94 (53.4)
Female	82 (46.6)
Smoking habits	
Never smoker	87 (49.4)
Smoker	89 (50.6)
Tumor differentiation	
Well	103 (58.5)
Moderate/poor	73 (41.5)
p‐TNM stage	
Stage I	118 (67.0)
Stage II	27 (15.3)
Stage III	31 (17.6)
Receiving adjuvant chemotherapy	
Yes	25 (14.2)
No	151 (85.8)
Vital status	
Alive	105 (59.7)
Lung cancer‐related death	51 (29.0)
Other causes of death	8 (4.5)
Unknown	12 (6.8)

Data are presented as No. (%).

p‐TNM, pathological TNM.

### IMMT expression in adenocarcinoma

**Figure 1:**
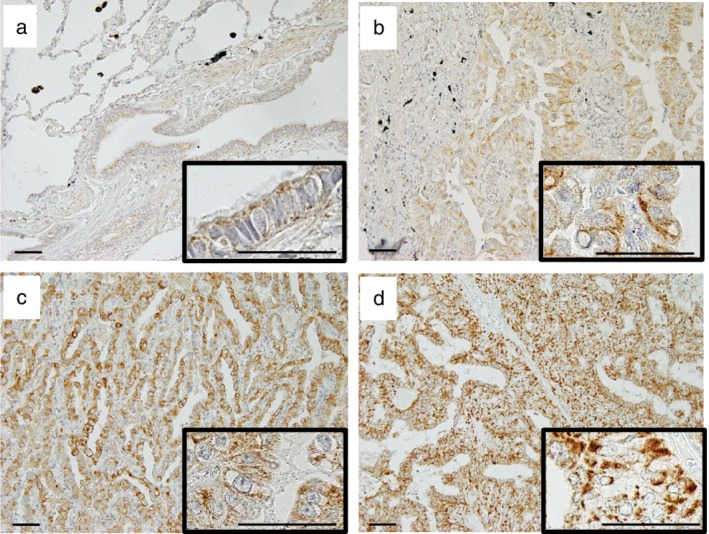
Expression of IMMT in lung adenocarcinomas. (**a**) Weakly granular staining of IMMT was observed in the cytoplasm of normal bronchial epithelial cells. In lung adenocarcinoma tissues, weakly granular cytoplasmic staining (**b**; immunoreactive score = 3), moderately granular cytoplasmic staining (**c**; immunoreactive score = 6) and marked granular cytoplasmic staining (**d**; immunoreactive score = 9) of tumor cells were observed (each inset shows high magnification). Scale bar = 50 μm.

In normal lung tissues, weakly cytoplasmic granular staining of IMMT was observed in bronchial epithelial cells (Fig 1a). In lung adenocarcinomas, weakly to marked cytoplasmic granular staining of IMMT was detected to varying degrees (Fig 1b–d). High‐IMMT expression was observed in 84 of 176 (47.4%) adenocarcinomas. No expression was observed in negative controls. The relationships between IMMT expression and clinicopathological characteristics are summarized in Table 2. High‐ IMMT expression was related to more advanced disease stage (stage II and III; *P* = 0.024), larger tumor size (>3cm; *P* = 0.002), and positive intratumoral vascular invasion (*P* < 0.001). There was no significant correlation between high‐IMMT expression and age, sex, smoking habits, tumor differentiation, intratumoral lymphatic invasion, pleural invasion, or the administration of adjuvant chemotherapy.

**Table 2:** Relationships between IMMT expression and clinicopathological parameters in lung adenocarcinoma

	IMMT expression			
Clinicopathological parameters	High (*n* = 84)	Low (*n* = 92)	Total	*P*‐value
Age				1.000
<65 years	45 (47.9)	49 (52.1)	94	
≥65 years	39 (47.6)	43 (52.4)	82	
Sex				1.000
Male	45 (47.9)	49 (52.1)	94	
Female	39 (47.6)	43 (52.4)	82	
Smoking habits				0.763
Never smoker	43 (49.4)	44 (50.6)	87	
Smoker	41 (46.1)	48 (53.9)	89	
Tumor differentiation				0.223
Well	45 (43.7)	58 (56.3)	103	
Moderate/poor	39 (53.4)	34 (46.6)	73	
p‐TNM stage				0.024
Stage I	49 (41.5)	69 (58.5)	118	
Stage II/III	35 (60.3)	23 (39.7)	58	
Tumor size				0.002
≤3 cm	37 (37.3)	62 (62.7)	99	
>3 cm	47 (61.0)	30 (39.0)	77	
Nodal status				0.085
N0	58 (43.9)	74 (56.1)	132	
N1/N2/N3	26 (59.1)	18 (40.9)	44	
Vascular invasion				0.000
No	45 (38.1)	73 (61.9)	118	
Yes	39 (67.2)	19 (32.7)	58	
Lymphatic invasion				0.733
No	63 (48.8)	66 (51.2)	129	
Yes	21 (44.7)	26 (55.3)	47	
Pleural invasion				0.144
No	53 (43.8)	68 (56.2)	121	
Yes	31 (56.4)	24 (43.6)	55	
Adjuvant chemotherapy				0.829
No	73 (48.3)	78 (51.7)	151	
Yes	11 (44.0)	14 (56.0)	25	

Data are presented as No. (%).

p‐TNM = pathological TNM.

Based on histological subtypes, high‐IMMT expression was detected in 11 of 23 acinar (47.8%), 27 of 58 lepidic (46.6%), nine of 11 micropapillary (81.8%), 29 of 64 papillary (45.3%), six of 13 solid (46.2%), and two of seven invasive mucinous (28.6%) adenocarcinomas. Micropapillary adenocarcinoma was associated with a significantly higher rate of high‐IMMT expression compared to that in lepidic (*P* = 0.032), papillary (*P* = 0.025), and invasive mucinous (*P* = 0.024) adenocarcinomas. Micropapillary adenocarcinoma also tended to have a higher rate of high‐IMMT expression than acinar disease (*P* = 0.060).

### Kaplan‐Meier estimate of survival in IMMT‐high and IMMT‐low patients

**Figure 2:**
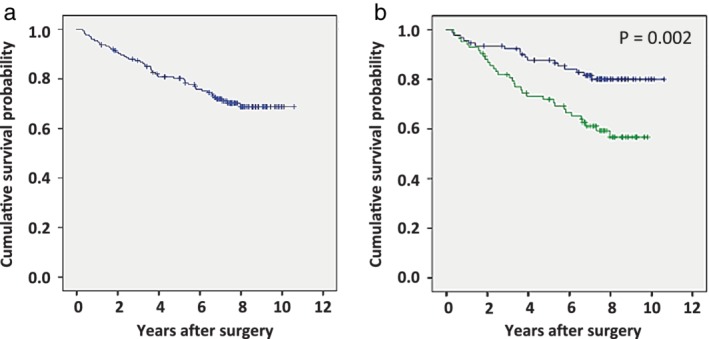
Cumulative survival of patients with lung adenocarcinoma estimated by the Kaplan‐Meier method. Patients with other causes of death and those lost to follow‐up were treated as censored cases. (**a**) In all 176 patients with resected lung adenocarcinoma, the five‐year cumulative survival probability was 80% and the median overall survival time (MST) was not determined () all, and () censored. (**b**) IMMT expression was significantly correlated with poorer survival in patients with lung adenocarcinoma. The five‐year cumulative survival probability for higher IMMT and lower IMMT expression groups were 72% and 87%, respectively, and the MST was not determined (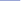) Low (N = 92), (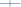) High (N = 84), (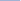) Low‐censored, and (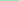) High‐consored.

All patients were included in the survival analysis. The overall follow‐up periods ranged from four to 129 months (median, 104 months) and the five‐year cumulative survival probability was 80% for all patients (Fig 2a). Because a cumulative survival probability of 50% was not reached, the overall median survival time was not determined. High‐IMMT expression group was significantly correlated with poorer survival compared to that in the low‐IMMT expression group of adenocarcinomas (*P* = 0.002; Fig 2b). Further, the five‐year survival probability was 72% and 87% for high‐ and low‐IMMT expression groups, respectively.

In addition, high‐IMMT expression group was also significantly correlated with poorer survival than those in low‐IMMT expression group in adenocarcinomas excluding the micropapillary subtype (*P* = 0.001, Fig S2).

### Effect of IMMT expression on survival based on univariate and multivariate analyses

For patients with adenocarcinoma, because survival was significantly correlated with IMMT expression, univariate and multivariate analyses were performed according to the Cox‐proportional hazard model to evaluate the effect of IMMT expression and other clinicopathological factors on survival based on 176 patients with lung adenocarcinoma. Univariate analysis indicated that p‐TNM stage (HR; 5.44; 95% confidence interval [CI], 3.02–9.80; *P* < 0.001), tumor differentiation (HR; 2.62; 95% CI, 1.48–4.62; *P* = 0.001), vascular invasion (HR; 3.82; 95% CI, 2.18–6.71; *P* < 0.001), lymphatic invasion (HR; 4.03; 95% CI, 2.30–7.06; *P* < 0.001), pleural invasion (HR; 2.88; 95% CI, 1.65–5.02; *P* < 0.001), adjuvant chemotherapy (HR; 3.22; 95% CI, 1.73–5.99; *P* = 0.001), and IMMT expression (HR; 2.40; 95% CI, 1.33–4.32; *P* = 0.003) were significant predictors of cancer‐specific survival (Table 3). Furthermore, IMMT expression and other clinicopathological variables including p‐TNM stage, tumor differentiation, vascular invasion, lymphatic invasion, pleural invasion, and adjuvant chemotherapy were entered into multivariate analysis using the Cox‐proportional hazards regression model. The results indicated that high‐IMMT expression was a significant independent predictor of poorer patient survival (HR; 1.99; 95% CI, 1.06–3.74; *P* = 0.031; Table 3).

**Table 3:** Univariate and multivariate analyses of the effect of IMMT expression on survival in patients with adenocarcinoma

	Univariate analysis			Multivariate analysis		
Factors	HR	95% CI	*P*‐value	HR	95% CI	*P*‐value
IMMT expression						
High vs. low	2.40	1.33–4.32	0.003	1.99	1.06–3.74	0.031
Age						
≥65 vs. <65	1.33	0.76–2.33	0.30	Not included in multivariable analysis		
Sex						
Male vs. female	0.99	0.57–1.73	0.98	Not included in multivariable analysis		
Smoking habits						
Smoker vs. never smoker	0.92	0.53–1.61	0.79	Not included in multivariable analysis		
p‐TNM stage						
Stage II/III vs. stage I	5.44	3.02–9.80	<0.001	2.11	1.02–4.34	0.042
Tumor differentiation						
Moderate/poorly vs. well	2.62	1.48–4.62	0.001	0.96	0.50–1.85	0.92
Vascular invasion						
Yes vs. no	3.82	2.18–6.71	<0.001	1.77	0.92–3.40	0.084
Lymphatic invasion						
Yes vs. no	4.03	2.30–7.06	<0.001	2.13	1.10–4.10	0.023
Pleural invasion						
Yes vs. no	2.88	1.65–5.02	<0.001	1.38	0.75–2.55	0.29
Adjuvant chemotherapy						
Yes vs. no	3.22	1.73–5.99	0.001	1.67	0.85–3.28	0.13

Analyses were performed using Cox proportional hazard regression.

HR, hazard ratio; p‐TNM, pathological TNM.

### *IMMT* mRNA expression levels and prognosis in lung adenocarcinoma patients

**Figure 3:**
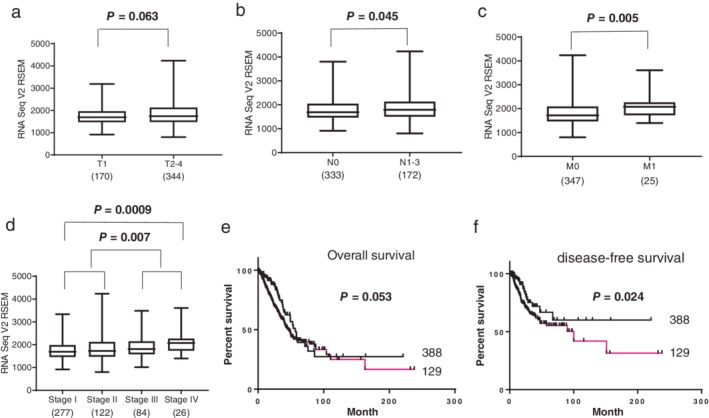
Prognostic significance of *IMMT* mRNA expression in lung adenocarcinoma based on TCGA data. *IMMT* mRNA was significantly higher in N1–3 than N0 (**b**; *P* = 0.045), M1 than M0 (**c**; *P* = 0.005), stage III–IV than stage I–II (**d**; *P* = 0.007), and stage IV than stage I (**d**; *P* = 0.0009). However, there was no association between T2‐4 and T1 (a; P = 0.063). The high‐*IMMT* mRNA expression group showed significantly poorer overall and disease‐free survival than the low‐*IMMT* mRNA group (**e**, **f**; *P* = 0.053 (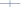) Low, and (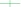) High and *P* = 0.024 (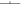) Low, and (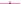) High, respectively).

To compare the present immunohistochemical data, we analyzed *IMMT* mRNA expression levels in lung adenocarcinoma patients using the TCGA database (Fig 3). Levels were significantly higher in N1–3 than N0 (Fig 3b, *P* = 0.045), M1 than M0 (Fig 3c, *P* = 0.005), stage III–IV than stage I–II (Fig 3d, *P* = 0.007), and stage IV than stage I (Fig 3d, *P* < 0.001) tumors. According to the log‐rank survival analysis, the high‐IMMT expression group showed significantly poorer overall survival and disease‐free survival than the low‐IMMT expression group of lung adenocarcinoma patients (*P* = 0.045 and *P* = 0.024, respectively).

### Transfection of *IMMT* siRNA and proliferation, migration, and invasion assays

IMMT protein was expressed at the same level in A549 and LC‐2/ad cell lines based on immunoblot analysis with our IMMT monoclonal antibody (Fig S3a). Thus, subsequent experiments used A549 cells. To investigate the role of IMMT in lung cancer cells, proliferation and transwell assays were performed after treated A549 cells treated with IMMT siRNA. Among the four *IMMT* siRNAs at different concentrations, siRNA #5 at 125 nM most effectively knocked down protein expression levels. Therefore, we used these conditions for the following study (Fig S3b). Compared to that in siControl‐transfected A549 cells, IMMT‐knockdown cells at 72 hours showed significantly reduced relative cell viability (*P* = 0.003; Fig S4a). Specifically, the relative cell viability of A549 cells treated with IMMT siRNA decreased to approximately 30% of siControl levels. With respect to migration and invasion capacities, no apparent differences were observed between siControl‐transfected and IMMT‐knockdown A549 cells (Fig S4b,c).

## Discussion

To date, no detailed study has examined the relationship between IMMT expression and clinicopathological parameters in any type of tumor. In the present study, we first confirmed that IMMT expression correlates with several clinicopathological parameters and poorer prognosis and is an independent prognostic factor for survival in patients with resected lung adenocarcinoma. The prognosis of patients with adenocarcinoma is principally correlated with the metastatic ability of tumor cells. The process of tumor metastasis consists of complex steps including those involving tumor cell migration followed by detachment from the primary tumor, invasion into the surrounding tissues, intravasation into blood or lymphatic vessels, dissemination in the hemolymphatic system, and extravasation at secondary sites.^13^ Based on clinicopathological characteristics, high‐IMMT expression was related to a more advanced stage of disease (stage II and III), larger tumor size (>3 cm), and intratumoral vascular invasion. These results suggest that IMMT expression might be important for the acquisition of growth and invasion capabilities in tumor cells, which subsequently results in poorer prognosis for patients with resected lung adenocarcinoma. John *et al*. reported that the downregulation of IMMT in HeLa cells, using specific small interfering RNA, led to a decreased growth rate and increased apoptosis rate in tumor cells, suggesting abnormal mitochondrial function.^6^ Recently, Madungwe *et al*. reported that IMMT knockdown induced cell death via apoptosis in an ALF–PARP‐dependent manner, which was associated with nuclear fragmentation and S phase cell cycle arrest.^14^ IMMT expression might also affect cellular proliferation and apoptosis in lung adenocarcinoma as previously described.^6^ The present study clarified that IMMT knockdown in A549 cells results in significantly reduced proliferation ability, confirming the results of a report of John *et al*. By profiling protein expression in tumor tissues based on disease recurrence within five years of follow‐up, Oshita *et al*. reported that IMMT is a biomarker that can predict tumor recurrence in early‐stage lung adenocarcinoma.^15^ These authors stated that proteins with prognostic significance detected in their analysis of early‐stage tumors were closely related to the initiation of invasion or adhesion during the metastatic process. Because the hypothesis that markers of mitochondrial biogenesis might have significant prognostic value for the early identification of high‐risk gastric cancer patients, Sotgia and Lisanti reported a survival analysis using the Kaplan‐Meier plotter based on an online public database. As a result, they clarified that *IMMT* and *VDAC3* mRNA levels have the best prognostic value for patients with gastric cancer.^16^

In this study, the micropapillary adenocarcinoma histological subtype showed a significantly increased rate of high‐IMMT expression compared to that with other histological subtypes. Adenocarcinoma with a micropapillary pattern is characterized by more frequent and prominent vascular invasion, a higher incidence and more advanced lymph node involvement, and a poorer prognosis compared to those with conventional papillary adenocarcinoma without the micropapillary pattern.^17^, ^18^, ^19^ Little is known about the factors and mechanisms underlying the more aggressive nature of this tumor subtype. However, IMMT might be involved in the aggressive nature of this tumor type. Accordingly, prognostic significance was found for patients with lung adenocarcinoma according to IMMT expression; as the sample size of patients is relatively small, a larger population will be needed to clarify the findings of the present study.

Adjuvant cisplatin‐based chemotherapy has been recommended to improve the survival of patients with completely resected stage II and IIIA adenocarcinoma, which has been associated with some improvement in the five‐year overall survival (ranging from 4% to 15%).^20^, ^21^ Traditionally, many researchers have focused on the interaction between mitochondrial dynamics and apoptosis; however, a recent study showed that many mitochondrial functions are associated with not only apoptosis but cancer progression, development, and chemoresistance.^22^ Invasive and metastatic cancer cells showed enhanced mitochondrial biogenesis, and clinical evidence has also supported the contention that increased mitochondrial biogenesis in invasive breast cancers is highly correlated with distant metastasis.^23^ It was inferred from these data that cancer cells utilize mitochondria‐mediated energy through the regulation of mitochondrial biogenesis for metastasis as well as survival. However, the exact underlying mechanism is still unknown. An *et al*. reported that a novel mitochondrial protein CHCM1/CHCHD6 directly interacts with IMMT and coordinately maintains the structural integrity of the mitochondrial cristae.^24^ Furthermore, they demonstrated that CHCM1/CHCHD6 knockdown affected cancer cell growth and enhanced chemosensitivity to anticancer drugs, whereas the increased exogenous expression of these markers desensitized cancer cells to these agents. In the present study, IMMT expression was significantly correlated with vascular invasion and a poorer prognosis in patients with adenocarcinoma. Data from An *et al*. 24 and the present study indicate that IMMT might also be involved in cancer cell growth, chemosensitivity, invasion, and metastasis. Although the function of IMMT with respect to invasive ability was not clarified in this study, the expression of this protein might be a useful marker to stratify high‐risk patients who should receive adjuvant chemotherapy. Further studies are needed to determine whether IMMT expression is a prognostic indicator that could help to select patients who might benefit from such regimens.

There are some limitations to our study. First, our study is a single‐institute retrospective cohort study. Second, with respect to the treatment of patients with postoperative recurrence, patients with EGFR mutations exhibit significantly longer survival than those with wild‐type EGFR when treated with EGFR‐TKIs.^25^ The relationship between IMMT expression and EGFR mutation status is unclear based on the results of the present study.

In summary, we report for the first time that IMMT expression is related to a more advanced stage of the disease, larger tumor size, and intratumoral vascular invasion in resected lung adenocarcinoma. Moreover, IMMT expression is associated with poorer prognosis and is an independent prognostic factor for survival in patients with this disease. Further studies are required to elucidate the biological function of IMMT in lung adenocarcinoma.

## Disclosure

No authors report any conflict of interest.

## Supporting information


**Appendix S1**: Supporting information

**Figure S1** Identification of antigen recognized by the KU‐Lu‐10 monoclonal antibody based on immunoprecipitation and mass spectrometry. (**a**) Proteins immunoprecipitated with the KU‐Lu‐10 antibody were separated by SDS‐PAGE and the gel was stained with the Zn‐staining kit (lane 1: molecular weight marker; lane 2: LCN1 lysate combined with KU‐Lu‐10 antibody and protein G; lane 3: KU‐Lu‐10 antibody combined with protein G; lane 4: LCN1 lysate combined with protein G; lane 5: LCN1 lysate). Lanes 3 and 4 are negative controls and the product immunoprecipitated with KU‐Lu‐10 was detected in lane 2. (**b**) Western blot analysis of immunoprecipitation samples and KU‐Lu‐10 hybridoma supernatant as the primary antibody. Negative controls are lanes 3 and 4, and the antigen immunoprecipitated with KU‐Lu‐10 antibody was detected in lane 2. The positive control is lane 5. Based on MALDI TOF/TOF‐MS analysis, the KU‐Lu‐10 antibody recognized IMMT [lane 2: LCN1 lysate combined with KU‐Lu‐10 antibody; lane 3: KU‐Lu‐10 antibody combined with protein G; lane 4: LCN1 lysate combined with protein G; lane5: LCN1 lysate]. (**c**) The KU‐Lu‐10 antibody reacted with recombinant N‐terminus FLAG‐GST‐labeled IMMT protein (FLJ92546AAAF) at 112 kDa, but not with the recombinant N‐terminus FLAG‐GST‐labeled Venus protein.

**Figure S2** Cumulative survival of patients with lung adenocarcinoma estimated by the Kaplan–Meier method. Patients with other causes of death and those lost to follow‐up were treated as censored cases. In all 165 patients with resected lung adenocarcinoma excluding the micropapillary subtype. IMMT expression was significantly correlated with poorer survival in patients with lung adenocarcinoma (*P* = 0.001). The five‐year cumulative survival probability for higher IMMT and lower IMMT expression groups were 67% and 82%, respectively.

**Figure S3** Transfection conditions of *IMMT* siRNA. (**a**) The IMMT protein was equally expressed in A549 and LC‐2/ad cell lines based on western blot analysis. To investigate the role for IMMT, A549 cells were treated with IMMT siRNA. (**b**) Among four IMMT siRNAs with different concentrations, siRNA #5 at 125 nM most effectively knocked down expression at the protein level.

**Figure S4** Transfection of *IMMT* siRNA and proliferation, migration, and invasion assays. Cell proliferation of IMMT‐knockdown A549 cells at 72 hours was significantly decreased to approximately 30% of levels observed with siControl A549 cells (*P* < 0.003) (**a**). No significant differences were observed in terms of migration and invasion in IMMT‐knockdown A549 cells compared to those in siControl cells (**b**, **c**).
