# Supplementary material for: Prognostic significance of IMMT expression in surgically‐resected lung adenocarcinoma
Source: Thorac Cancer. 2019 Oct 3;10(11):2142–51. doi: 10.1111/1759-7714.13200 (PMC6825906; doi:10.1111/1759-7714.13200)
Supplement: Supplementary file 1 — Appendix S1: Supporting information [file TCA-10-2142-s001.pptx]

## Slide 1
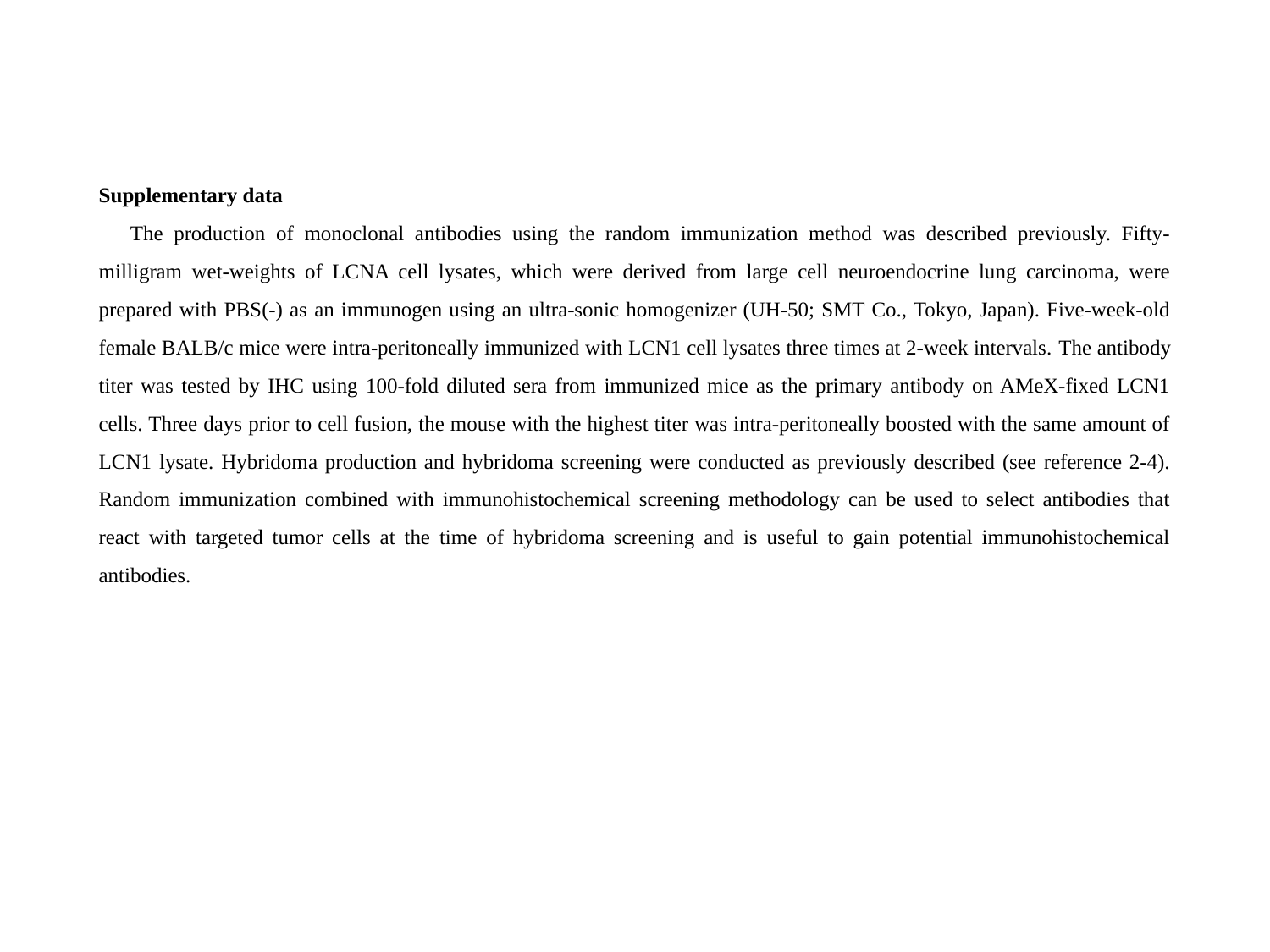

Supplementary data
　The production of monoclonal antibodies using the random immunization method was described previously. Fifty-milligram wet-weights of LCNA cell lysates, which were derived from large cell neuroendocrine lung carcinoma, were prepared with PBS(-) as an immunogen using an ultra-sonic homogenizer (UH-50; SMT Co., Tokyo, Japan). Five-week-old female BALB/c mice were intra-peritoneally immunized with LCN1 cell lysates three times at 2-week intervals. The antibody titer was tested by IHC using 100-fold diluted sera from immunized mice as the primary antibody on AMeX-fixed LCN1 cells. Three days prior to cell fusion, the mouse with the highest titer was intra-peritoneally boosted with the same amount of LCN1 lysate. Hybridoma production and hybridoma screening were conducted as previously described (see reference 2-4). Random immunization combined with immunohistochemical screening methodology can be used to select antibodies that react with targeted tumor cells at the time of hybridoma screening and is useful to gain potential immunohistochemical antibodies.
