# Supplementary material for: Prognostic significance of IMMT expression in surgically‐resected lung adenocarcinoma
Source: Thorac Cancer. 2019 Oct 3;10(11):2142–51. doi: 10.1111/1759-7714.13200 (PMC6825906; doi:10.1111/1759-7714.13200)
Supplement: Supplementary file 2 — Figure S1 Identification of antigen recognized by the KU‐Lu‐10 monoclonal antibody based on immunoprecipitation and mass spectrometry. (a) Proteins immunoprecipitated with the KU‐Lu‐10 antibody were separated by SDS‐PAGE and the gel was stained with the Zn‐staining kit (lane 1: molecular weight marker; lane 2: LCN1 lysate combined with KU‐Lu‐10 antibody and protein G; lane 3: KU‐Lu‐10 antibody combined with protein G; lane 4: LCN1 lysate combined with protein G; lane 5: LCN1 lysate). Lanes 3 and 4 are negative controls and the product immunoprecipitated with KU‐Lu‐10 was detected in lane 2. (b) Western blot analysis of immunoprecipitation samples and KU‐Lu‐10 hybridoma supernatant as the primary antibody. Negative controls are lanes 3 and 4, and the antigen immunoprecipitated with KU‐Lu‐10 antibody was detected in lane 2. The positive control is lane 5. Based on MALDI TOF/TOF‐MS analysis, the KU‐Lu‐10 antibody recognized IMMT [lane 2: LCN1 lysate combined with KU‐Lu‐10 antibody; lane 3: KU‐Lu‐10 antibody combined with protein G; lane 4: LCN1 lysate combined with protein G; lane5: LCN1 lysate]. (c) The KU‐Lu‐10 antibody reacted with recombinant N‐terminus FLAG‐GST‐labeled IMMT protein (FLJ92546AAAF) at 112 kDa, but not with the recombinant N‐terminus FLAG‐GST‐labeled Venus protein. [file TCA-10-2142-s002.pptx]

## Slide 1
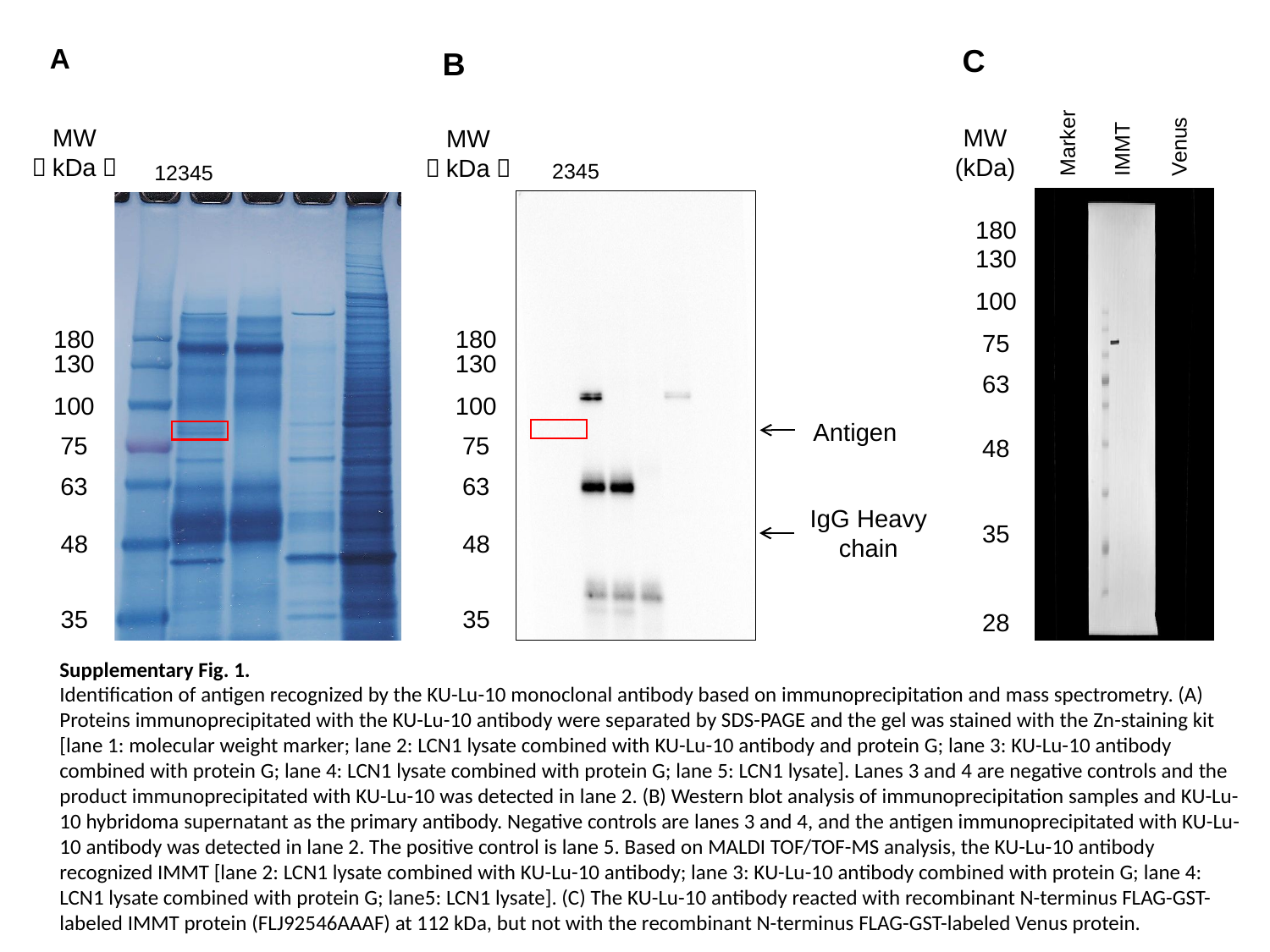

A
C
B
Venus
IMMT
Marker
MW
(kDa)
MW
（kDa）
MW
（kDa）
2345
12345
180
130
100
180
180
75
130
130
63
100
100
Antigen
75
75
48
63
63
IgG Heavy chain
35
48
48
35
35
28
Supplementary Fig. 1.
Identification of antigen recognized by the KU-Lu-10 monoclonal antibody based on immunoprecipitation and mass spectrometry. (A) Proteins immunoprecipitated with the KU-Lu-10 antibody were separated by SDS-PAGE and the gel was stained with the Zn-staining kit [lane 1: molecular weight marker; lane 2: LCN1 lysate combined with KU-Lu-10 antibody and protein G; lane 3: KU-Lu-10 antibody combined with protein G; lane 4: LCN1 lysate combined with protein G; lane 5: LCN1 lysate]. Lanes 3 and 4 are negative controls and the product immunoprecipitated with KU-Lu-10 was detected in lane 2. (B) Western blot analysis of immunoprecipitation samples and KU-Lu-10 hybridoma supernatant as the primary antibody. Negative controls are lanes 3 and 4, and the antigen immunoprecipitated with KU-Lu-10 antibody was detected in lane 2. The positive control is lane 5. Based on MALDI TOF/TOF-MS analysis, the KU-Lu-10 antibody recognized IMMT [lane 2: LCN1 lysate combined with KU-Lu-10 antibody; lane 3: KU-Lu-10 antibody combined with protein G; lane 4: LCN1 lysate combined with protein G; lane5: LCN1 lysate]. (C) The KU-Lu-10 antibody reacted with recombinant N-terminus FLAG-GST-labeled IMMT protein (FLJ92546AAAF) at 112 kDa, but not with the recombinant N-terminus FLAG-GST-labeled Venus protein.
