# Supplementary material for: Prognostic significance of IMMT expression in surgically‐resected lung adenocarcinoma
Source: Thorac Cancer. 2019 Oct 3;10(11):2142–51. doi: 10.1111/1759-7714.13200 (PMC6825906; doi:10.1111/1759-7714.13200)
Supplement: Supplementary file 3 — Figure S2 Cumulative survival of patients with lung adenocarcinoma estimated by the Kaplan–Meier method. Patients with other causes of death and those lost to follow‐up were treated as censored cases. In all 165 patients with resected lung adenocarcinoma excluding the micropapillary subtype. IMMT expression was significantly correlated with poorer survival in patients with lung adenocarcinoma (P = 0.001). The five‐year cumulative survival probability for higher IMMT and lower IMMT expression groups were 67% and 82%, respectively. [file TCA-10-2142-s003.pptx]

## Slide 1
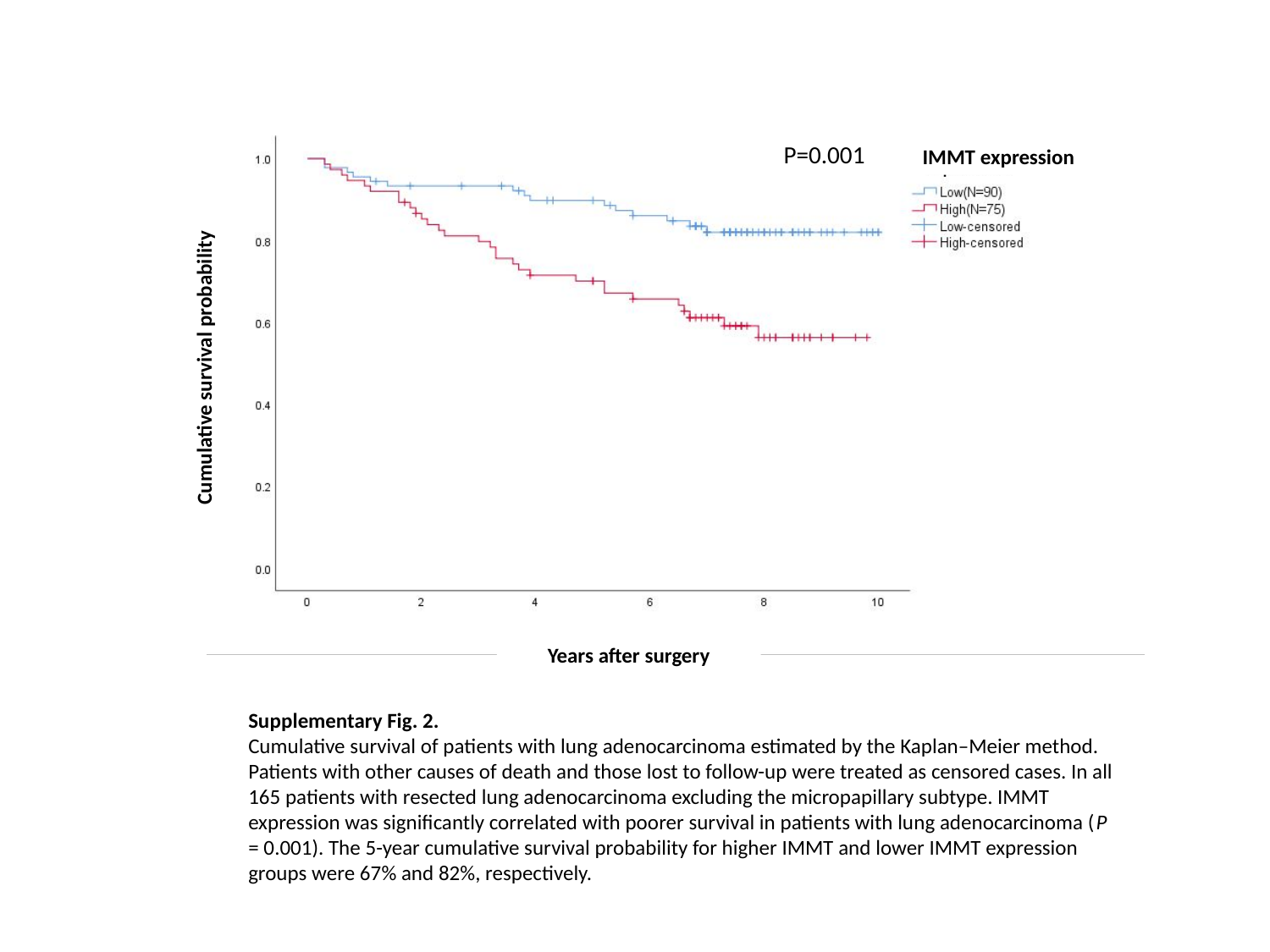

P=0.001
Cumulative survival probability
IMMT expression
B
Cumulative survival probability
P = 0.002
Years after surgery
Supplementary Fig. 2.
Cumulative survival of patients with lung adenocarcinoma estimated by the Kaplan–Meier method. Patients with other causes of death and those lost to follow-up were treated as censored cases. In all 165 patients with resected lung adenocarcinoma excluding the micropapillary subtype. IMMT expression was significantly correlated with poorer survival in patients with lung adenocarcinoma (P = 0.001). The 5-year cumulative survival probability for higher IMMT and lower IMMT expression groups were 67% and 82%, respectively.
