# Supplementary material for: Prognostic significance of IMMT expression in surgically‐resected lung adenocarcinoma
Source: Thorac Cancer. 2019 Oct 3;10(11):2142–51. doi: 10.1111/1759-7714.13200 (PMC6825906; doi:10.1111/1759-7714.13200)
Supplement: Supplementary file 4 — Figure S3 Transfection conditions of IMMT siRNA. (a) The IMMT protein was equally expressed in A549 and LC‐2/ad cell lines based on western blot analysis. To investigate the role for IMMT, A549 cells were treated with IMMT siRNA. (b) Among four IMMT siRNAs with different concentrations, siRNA #5 at 125 nM most effectively knocked down expression at the protein level. [file TCA-10-2142-s004.pptx]

## Slide 1
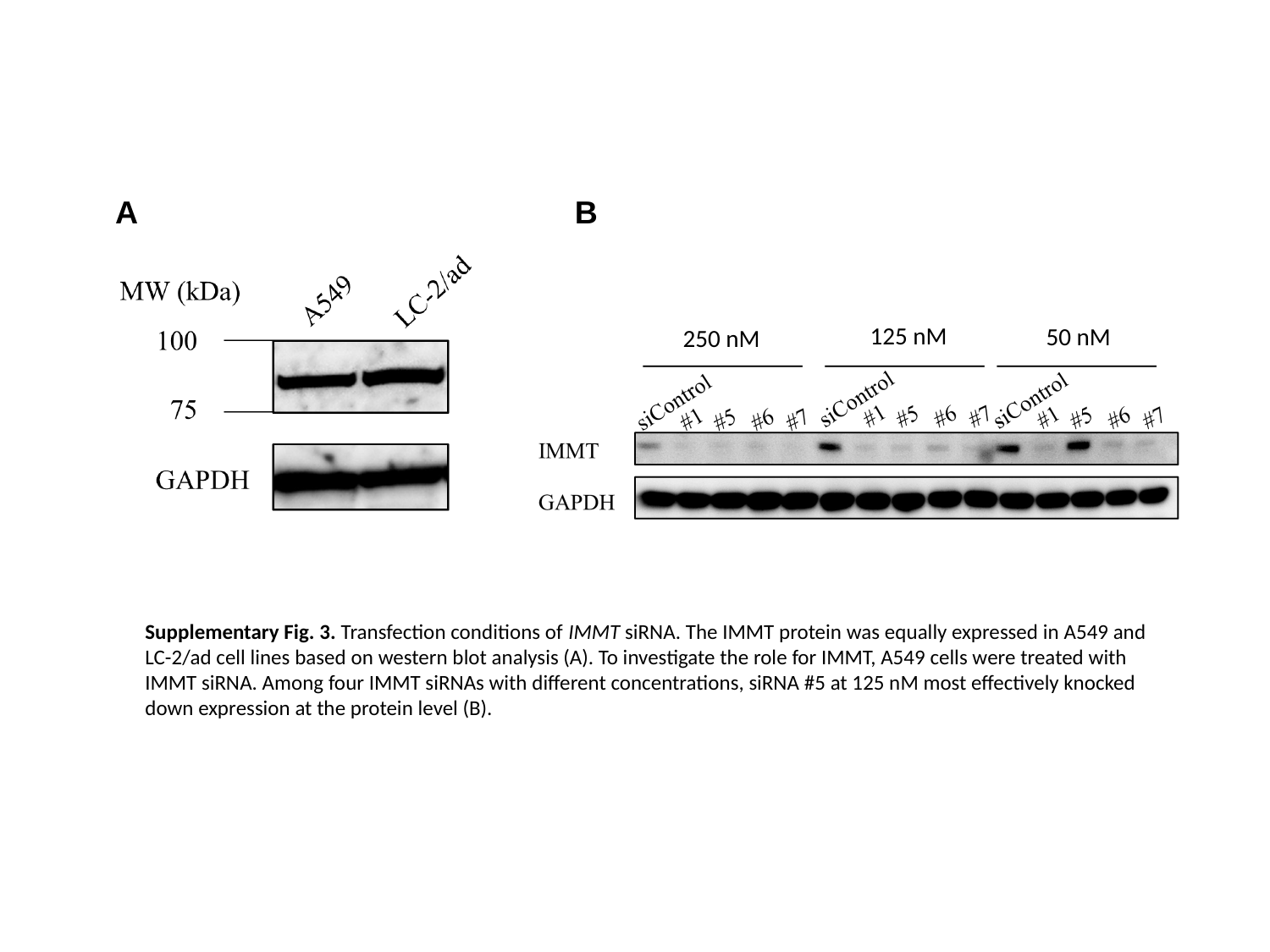

A
B
125 nM
50 nM
250 nM
Supplementary Fig. 3. Transfection conditions of IMMT siRNA. The IMMT protein was equally expressed in A549 and LC-2/ad cell lines based on western blot analysis (A). To investigate the role for IMMT, A549 cells were treated with IMMT siRNA. Among four IMMT siRNAs with different concentrations, siRNA #5 at 125 nM most effectively knocked down expression at the protein level (B).
