# Supplementary material for: Prognostic significance of IMMT expression in surgically‐resected lung adenocarcinoma
Source: Thorac Cancer. 2019 Oct 3;10(11):2142–51. doi: 10.1111/1759-7714.13200 (PMC6825906; doi:10.1111/1759-7714.13200)
Supplement: Supplementary file 5 — Figure S4 Transfection of IMMT siRNA and proliferation, migration, and invasion assays. Cell proliferation of IMMT‐knockdown A549 cells at 72 hours was significantly decreased to approximately 30% of levels observed with siControl A549 cells (P < 0.003) (a). No significant differences were observed in terms of migration and invasion in IMMT‐knockdown A549 cells compared to those in siControl cells (b, c). [file TCA-10-2142-s005.pptx]

## Slide 1
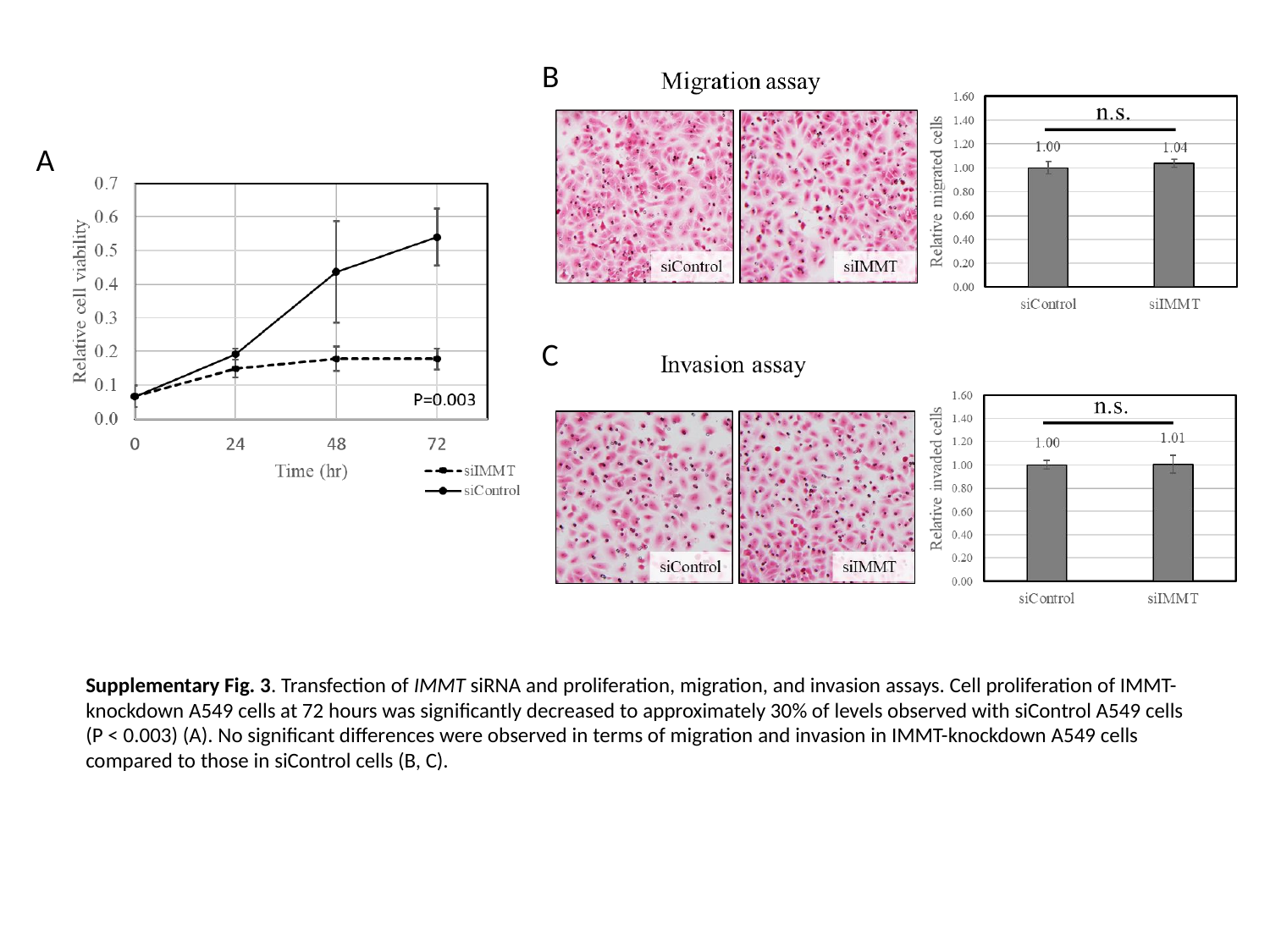

B
A
C
Supplementary Fig. 3. Transfection of IMMT siRNA and proliferation, migration, and invasion assays. Cell proliferation of IMMT-knockdown A549 cells at 72 hours was significantly decreased to approximately 30% of levels observed with siControl A549 cells (P < 0.003) (A). No significant differences were observed in terms of migration and invasion in IMMT-knockdown A549 cells compared to those in siControl cells (B, C).
